# Is benefit sharing with research participants lawful in South Africa? An unexplored question in the governance of genomics research

**DOI:** 10.1093/jlb/lsad018

**Published:** 2023-06-28

**Authors:** Donrich Thaldar, Bonginkosi Shozi

**Affiliations:** School of Law, University of KwaZulu-Natal, Durban, South Africa; Petrie-Flom Center for Health Law Policy, Biotechnology, and Bioethics, Harvard Law School, Cambridge, MA, USA; Institute for Practical Ethics, University of California San Diego, San Diego, CA, USA

## Abstract

Despite advocacy in favour of benefit sharing with research participants in genomics research that is conducted in South Africa, there has been little critical legal engagement with this concept. That is what this article provides by posing the hitherto unexplored—but foundational—question: Is benefit sharing with research participants lawful in South Africa? The answer is clearly ‘no’. South African law provides that it is unlawful to provide any financial or other reward to research participants for donating biospecimens—except for reimbursement of reasonable costs incurred. Accordingly, benefit sharing would be unlawful. The ramifications of this conclusion are far-reaching. Most pertinently, should any benefit-sharing agreements with research be put into practice, such agreements would be unenforceable and would expose all parties involved—including foreign collaborators—to criminal prosecution. The solution for proponents of benefit sharing in South Africa would be to lobby the South African government to revise the relevant law. However, as long as the law remains as it currently is, institutions and individuals all over the world who are involved in genomics research in South Africa would be well advised to comply with the law by not engaging in benefit sharing with research participants.

## I. INTRODUCTION

Samples of human biological material (also known as ‘biospecimens’^1^) are critical to the development of human genomic health research at a global level. Data derived from genetic analysis of biospecimens, often forming parts of large repositories (known as ‘biobanks’), are integral to growing our understanding of how to avoid disease, how diseases function, and the development of more effective pharmaceutical interventions.^2^ Typically, the individuals from whom biospecimens are acquired do not get anything in return for the research participation. In recent years, however, concerns have been raised about the extent to which this is just and equitable in circumstances where research participants are individuals or community groups from historically disadvantaged backgrounds. This is on the premise that such individuals are often excluded from partaking in the fruits of scientific research, including the gains that may arise from the research projects using their biospecimens. For example, Bedeker et al. concisely elucidated the crux of this concern by remarking that, ‘individual research participants in the Global South rarely experience benefits from the research in which they participate’ and thus argue for research involving the biospecimens of individuals in the Global South to include an arrangement for benefit sharing.^3^

Benefit sharing is argued by proponents to be a necessary part of the governance of health research involving human biospecimens, on the basis that this policy is a vehicle for ameliorating distributive inequality in the enjoyment of the fruits of genomic health research, typically through the redistribution of profits back to research participants.^4^ While calls have been made for the inclusion of benefit-sharing arrangements with research participants in the South African context,^5^ there has not been critical legal engagement with this concept. That is what this article provides. We pose the hitherto unexplored question: Is benefit sharing with research participants lawful in South Africa?

It is important to answer this legal question to ensure that stakeholders involved in genomic health research in South Africa do not unwittingly enter into arrangements that violate the law, as this could lead to outcomes that are contrary to the interests of researchers and research participants.

## II. OVERVIEW OF LITERATURE ON BENEFIT SHARING

### II.A. What Is Benefit Sharing?

Benefit sharing, while a popular concept, is one that lacks a universally recognized definition. This is because many of its proponents discuss benefit sharing by making a case for its necessity, without critical intellectual engagement with technical questions such as what it means.^6^ One oft-cited definition by Schroeder endeavors to sum up the concept as follows:

‘Benefit sharing is the action of giving a portion of advantages/profits derived from the use of human genetic resources to the resource providers to achieve justice in exchange, with a particular emphasis on the clear provision of benefits to those who may lack reasonable access to resulting healthcare products and services without providing unethical inducements.’^7^

As the above definition alludes to, there is a normative claim embedded in arguments for benefit-sharing arrangements, which may be expressed as follows: *insofar as advantages are to be gained from research on biospecimens, justice and equality require that the persons that enable access to the biospecimens are owed some share of these gains*.^8^ The underlying premise that informs this normative claim is that research involving underprivileged and marginalized persons is currently unjust and inequitable as these persons are deprived access to these gains; therefore, benefit-sharing arrangements are necessary for these research projects to be just and equitable.^9^ Put differently, advocates regard benefit sharing as an indispensable means of securing an equitable distribution of the fruits of scientific research. One of the most prominent examples of this is the Human Genome Organisation (HUGO) Ethics Committee’s *Statement on Benefit Sharing*, in which it is explicitly stated that its aim is to ‘distribute profits that may accrue to commercial enterprises, governments, or academic institutions on the basis of the participation of particular communities’.^10^ Accordingly, while humanity as a whole may enjoy the fruits of research involving underprivileged and marginalized persons that advance human health, given that underprivileged and marginalized persons are often excluded from the distribution of the fruits of science and technology even if they contribute to it, interventions are necessary to ensure that these persons receive their fair share of the benefits in a targeted and proximate way.^11^

### II.B. What Are Benefits?

It is trite that in bioethics broadly, the term ‘benefits’ typically refers to acts that advance the interests (ie that which is objectively good for a person) and well-being (ie one’s subjective experience of the good) of a person. But this general definition sheds little light on what proponents of benefit sharing mean when they speak of sharing benefits, thus it is necessary to interrogate their use of the concept more closely.

From the various contexts within which the term has been used in the context of benefit sharing, it is possible to differentiate between at least two general categories of benefits: benefits as sharing the fruits of scientific research and benefits as direct advancement of interests. The first category conceives of benefits as the fruits of research, and typically entails sharing these benefits with those who contribute to the research by providing the researchers access to biospecimens. One can spot this conceptualization of benefit sharing in the definition put forward by Schroeder above, which suggests that a benefit is a share of the ‘advantages’ derived from the research project, that is shared with the ‘resource providers’. Put differently, benefits in this first category are outputs from a research project that may advance the interests or well-being of those make access to the biospecimens possible. These may include access to the product, healthcare intervention, or knowledge resulting from the research project for the research participant or community;^12^ a share of the profits being provided to research participants and their communities;^13^ having the results deriving from the research made available to the research participants and the community;^14^ and giving acknowledgement^15^ or recognition^16^ to research participants and their communities in respect of any outputs.

Of note is that with this category of benefit sharing, the benefit’s existence is one that is conditional on the research actually bearing fruit. The benefit may therefore never accrue, and if it does, the benefit only comes into existence at some point in the future. An important implication of this is that if a research participant is among those who are to be recipients of benefits, his or her interests or well-being is not advanced in the short term by their participation in the research project. It may, however, be the case that his or her interests are substantially advanced in the long term, such as where his or her financial interests are advanced by receiving a share of the profits deriving from the research project, or their well-being is advanced by having access to a life-changing healthcare intervention.

The second category of benefits entails advancing the well-being of those who provide access to biospecimens directly, and thus conceives of benefits as anything that advances the interests and well-being of research participants, their communities, and/or any other entity that enables access to biospecimens. This general category is to be differentiated from the first in that it does not entail researchers sharing potentially speculative future benefits deriving from a specific research project. Rather, it entails researchers undertaking projects intended to advance the interests and well-being of a category or group of persons, regardless of the outcome of a particular research project. This may include providing medical care to research participants and their communities;^17^ developing infrastructure in the area where the gathering of biospecimens takes place;^18^ providing employment opportunities and/or skills development programs for people who live in the area where the gathering of biospecimens takes place;^19^ and facilitating research capacity development for scientists who are from, or live in, the polity where the gathering of biospecimens takes place.^20^

It is often noted that what determines an appropriate benefit ‘depends on needs, values, priorities and cultural expectations’.^21^ In simple terms, this is something to be determined by negotiation between the researchers and the stakeholders with whom they are required to engage for the purposes of receiving approval to conduct research. This may be via a national research ethics committee, a community representative, or, in some cases, even the individual research participant. Identifying the appropriate stakeholder will depend on several factors including the regulatory framework in the area where the research is to be conducted, and the kind of research that is to be carried out—as this influences who may be entitled to receive benefits.

### II.C. Who Ought to Be the Recipients of Benefit Sharing?

Benefit sharing is not intended to apply in all cases. Rather, advocates of benefit sharing argue that it only applies in the context of research where considerations of justice and equity necessitate it. This is most commonly the case where a research project involves a group of individuals, for the obvious reason that genomic health research projects often require large collections of biospecimens. The number of research participants that biospecimens are derived from will however vary significantly—from a few dozen to potentially thousands. The common factors among these research participants may also vary significantly. They may share close genetic ties (such as an ethnic community) or perhaps even just a single genetic trait, such as a mutation.

It is usually the case that benefit sharing is advocated for in respect of research on groups which are described as ‘communities’. Note, however, that the term ‘community’ is used quite loosely in the literature on benefit sharing. For instance, the HUGO *Statement on Benefit Sharing* describes two kinds of communities that may be involved in benefit sharing:^22^ communities of origin (such as indigenous communities and family groups) and communities of circumstance (such as interest groups and voluntary associations). The former (communities of origin) are what most people understand by the term, and these kinds of communities have received the most attention in the discourse on benefit sharing. As Bedeker et al. acknowledge, benefit sharing traditionally has a ‘microlevel’ focus, which is concerned with ‘research participants and patients, their families and immediate communities where research is taking place’.^23^ The reason for this, as alluded to above, is that benefit sharing is premised on the normative claim that those who provide the genetic resources that enable research are ‘owed’ something.

The case of individuals, family groups, or ethnic communities being entitled to benefits for ‘their’ biospecimens and associated data is one that is often made—despite how difficult it is to justify.^24^ It is also widely contended that other persons or entities, besides the research participants and their kin, also have such an entitlement due to their relationship with the persons from whom the biospecimens derive. For instance, Sheremeta and Knoppers identify governments, non-government organizations’, and academic institutions as possible beneficiaries of benefit-sharing arrangements.^25^ As with communities, the status of institutions as beneficiaries is premised on their having some entitlement to the biospecimens. For example, in the case of governments, this might be because the community in question are all members of their polity, and thus the government has authority over the use of their biospecimens.

It is important to note that while the literature frequently refers to community groups as beneficiaries, individuals can also be beneficiaries. The term ‘benefit sharing’ has, in several instances, been used by proponents of the concept to describe circumstances where benefits may accrue to individuals, pursuant to them donating their biospecimens.^26^ In other words, an individual is purportedly entitled to benefits deriving from a particular research project only if he or she agrees to be a research participant—such that his or her status as a beneficiary is subject to him or her donating biospecimens to the research project. For example, Sheremeta and Knoppers make the case that ‘research participants in externally sponsored research are also entitled to health care services, treatment of any ensuing injury and to benefit from services related to the interventions or products developed’.^27^ Accordingly, those propositioned for such research will be aware that the benefit-sharing arrangements will only make these benefits available to those who donate their biospecimens.

A discussion of the nature and validity of entitlements to benefit sharing is beyond the scope of this paper. Suffice to say, whether one agrees with the existence of such entitlements or not, arrangements in terms of which there are benefits to be shared are intended to materially advance the interests or well-being of a specific individual by virtue of him or her providing biospecimens for use in research fall, within the definition of benefit sharing. In addition, it is also clear that some benefits (such as services that are made available to a patient interest group of which a research participant is a member), which are not aimed at a specific individual, may nevertheless also meaningfully advance the interests and well-being of a specific individual by virtue of him or her providing biospecimens for use in research. It is important to note how benefit-sharing arrangements may advance the interests and well-being of individuals by virtue of their status as research participants, as it is here that the legal question arises.

## III. ANALYSIS OF SOUTH AFRICAN HEALTH LAW

The statutory framework for regulating human research participation in South Africa is founded in a paradigm of altruism*.*^28^ Core to this statutory framework that enforces altruism is section 60(4) of the National Health Act, which provides as follows:

‘It is an offence for a person—(a) who has donated tissue, a gamete, blood or a blood product to receive any form of financial or other reward for such donation, except for the reimbursement of reasonable costs incurred by him or her to provide such donation.’

For ease of reading, we collectively refer to the quartet of tissue, gametes, blood, and blood products as *biospecimens*. For the purposes of this article, it is not necessary to explore the definition of *tissue* in detail. Suffice to say, it is defined more broadly than its ordinary meaning to include biological material such as body fluids, which are often used in genetics research. However, *tissue* does not include material, such as cell lines and DNA that are *derived* from a tissue sample. By contrast, all derivatives of blood are included in the definition of *blood products*. Accordingly, when we refer to *biospecimens*, it generally includes only the biological samples in the form as collected from research participants, with the exception that all derivatives of blood are included.

What is the import of the provision quoted above? It provides that persons who donated biospecimens for research—research participants—may not receive any form of financial or other reward, with only one exception: reimbursement of reasonable costs incurred. The broad formulation of the ban—‘any form of financial of other reward’—is worth noting. As the word *reward* is not defined in the Act, it takes its ordinary meaning, which is ‘[a] thing given in recognition of service, effort, or achievement’.^29^ The word *thing* in turn does not include only a physical object, but also an action, event, thought, or utterance.^30^ Therefore, any physical object, action, event, thought, or utterance given to a research participant in recognition of his or her donation of a biospecimen is unlawful—except for reimbursement of reasonable costs incurred. This suggests that benefit sharing, which entails the advancement of the interests and well-being of research participants, is a reward—and is therefore unlawful.

### III.A. Purposive Interpretation

Is this truly the *purpose* of this section of the South African National Health Act—to make benefit sharing *unlawful*? Note that South African law takes a purposive approach to the interpretation of statutes, which entails considering the purpose of a legal provision by looking not only at the words used but also considering the provision in the broader context of the statute as a whole. The purpose of the Act, as stated in its preamble, is to ‘establish a health system based on … principles of equity, efficiency, sound governance, internationally recognised standards of research and a spirit of enquiry and advocacy which encourages participation’. An argument can therefore be constructed that many of these principles, in particular (i) equity, (ii) internationally recognized standards of research, and (iii) encouragement of participation in research, all point to *allowing* benefit sharing and not outlawing it. However, this argument is premised on (i) the ethical claim that benefit sharing is more equitable than altruism, (ii) the factual claim that international standards about human participants in research generally prescribe benefit sharing, and (iii) the sociological claim that benefit sharing is better than altruism in encouraging research participation. Proving these premises would be a steep hill to climb:

(i) The ethical claim that benefit sharing is more equitable than altruism is contested.
(ii) While some international ethics instruments call for benefit sharing with research participants, it is a well-established international standard for human participation in research that such participation must be voluntary and not unduly enticed. This is especially the case in a country such as South Africa, where many people are poor, and where it can be argued that any kind of reward for research participation would constitute undue enticement that would compromise the internationally recognized principle that research participation must be altruistic.
(iii) The sociological claim that benefit sharing is better than altruism in encouraging research participation would require empirical data presented by expert testimony, which would be subjected to lawyers’ scrutiny. Important in this context is to consider the existence of altruistic avenues to encourage participation in research, such as awareness campaigns to socially construct participation in research as a civic virtue, and whether such altruistic avenues have been assessed through large-scale sociological studies.

Even if one can prove, for the sake of argument, that any or all of the principles (i) to (iii) above favor benefit sharing, one would still be confronted with the clear and unambiguous language of section 60(4), which does not allow for alternative interpretations. Moreover, the purpose of section 60(4)—to uphold a regime of *altruism* in respect of dealing with human biospecimens in the research context—is evident from this clear and unambiguous language. By using the language ‘any form of financial or *other reward*’, it is clear that the aim of this provision is to prohibit individuals from having their interests and well-being advanced in any way (financial or otherwise) by virtue of being a biospecimen donor. Evidently, at the time when the legislature stated its allegiance with principles (i) to (iii), the legislature viewed these principles as aligned with *altruism* in respect of dealing with human biospecimens in the research context—and did not intend to permit deviations from altruism, such as benefit sharing. Accordingly, both a plain reading of the language of section 60(4) and a purposive reading of the provision confirm that benefit sharing with research participants, if practiced in South Africa, would be unlawful.

### III.B. Altruism Is Compatible with Benefits to Society in General

The following counterargument can be constructed: The intention of health research is to develop new and better healthcare products and services. Such outcome is beneficial for society in general. Accordingly, even if an individual research participant does not benefit *directly* from such outcome, the research participant, as a member of society, benefits *indirectly*. It would clearly be absurd—and practically impossible—to suggest that the research participant should somehow not benefit from such fruits of research. But if this is so, does it not mean that the prohibition on receiving a reward as per section 60(4) is untenable?

The answer is no. The prohibition on receiving a reward as per section 60(4) is specific to receiving a reward for donating a biospecimen. In other words, what is unlawful is to receive a reward by virtue of being a research participant in a research project that involves the collection of biospecimens. By contrast, if one enjoys the fruits of research that become available to society in general, one does so by virtue of being a member of society—not by virtue of being a research participant in the project that yielded such fruits. Accordingly, enjoyment of the fruits of research that become available to society in general (for example a new medicine that is accessible through the healthcare system) is not a reward as contemplated in section 60(4).

Next, we consider a possible legal strategy that pro-benefit-sharing activists might consider to challenge the current legal position: namely human rights litigation that impugns section 60(4).

### III.C. Constitutionality Analysis

In South Africa, the judiciary is not limited to just interpreting statutes; they also have the power of judicial review of statutes. Thus, possible a possible legal strategy would be to accept that the extant law outlaws benefit sharing, but to challenge its constitutionality on human rights grounds. We now consider the merits of such a strategy. An argument can be constructed along the following lines: By allowing everybody involved in research—except the research participants—to benefit in some way from research, a political value judgment is made that research participants as a class of persons are *not worthy* of reward, even if they are the *conditio sine qua non* of the entire research endeavor. This relegates them to an inferior position that infringes their right to dignity (Dignity is an enforceable right in South African law.^31^ It has been interpreted to have several dimensions, of which *self-worth* is at the core.)^32^^,^^33^ If this argument is accepted by the court—which is all but guaranteed, but possible if well argued—the legal onus will shift to the South African government to *justify* the limitation imposed on the right to dignity by section 60(4). Here, the first step would be for the government to identify one or more *legitimate government purposes* served by section 60(4). These could include (a) preventing the commercialization of the human body, (b) preventing exploitation of vulnerable persons, and (c) promoting civic virtue. Government could further argue that these purposes in turn serve important constitutional values, including dignity and building a caring society,^34^ and that any limitation of rights that may be imposed by section 60(4) is outweighed by the legitimate government purposes served by section 60(4). Accordingly, the human rights litigation strategy has its weaknesses from the perspective of creating a legal opening for practicing benefit sharing in South Africa.

Such a lawsuit may, however, serve the extra-curial strategic purpose of political agenda-setting. To explain: A human rights lawsuit instituted against the South African government may start receiving media attention and would therefore place political pressure on government to seriously consider the issue of benefit sharing. Government would need to decide right at the outset whether to actively oppose the lawsuit or simply to abide by the decision of the court. Government may even—if it considers that there is merit in the lawsuit—request the party that filed the lawsuit to suspend the litigation to give it time to prepare amendments to the impugned legislation. Given that an amendment to the National Health Act is not within the power of the executive branch of government—this will have to be done by parliament—the government will not be able to settle the lawsuit on the basis of delivering the desired amendment. However, what is within the executive’s power is to prepare an amendment and bring it before parliament within a certain timeframe. In other words—political agenda-setting.

### III.D. The Criminal Law

As we have concluded in our analysis above, extant law in South Africa prohibits any form of reward for giving one’s biological samples for research, and therefore effectively outlaws benefit sharing for research participants, with the possible exception of small non-monetary and symbolic types of benefit sharing, such as acknowledgement in publications. Moreover, the National Health Act provides in section 60(5) that such conduct is not merely unlawful, but also *criminal*, and subject to a fine or imprisonment for a period not exceeding 5 years or to both. This may not be pleasant reading to proponents of benefit sharing, but the criminal law implications of implementing benefit sharing measures under the current legal regime is a South African reality. It is naïve and downright dangerous to ignore this reality.

First, it is important to understand that it is not merely the research participant receiving a reward who will be guilty of criminal conduct, but also all other persons who aided and abetted the crime. In other words, anyone who offers a reward or allows the reward to be made will be guilty as accessories to the crime. Furthermore, even just planning to offer someone a reward for donating a biospecimen would be conspiring to commit a crime, and hence it would be a prosecutable crime *per se.* The National Prosecuting Authority (NPA) can elect to prosecute only certain wrongdoers, and not others. For example, the NPA can decide not to press charges against research participants, but only against the researchers, ethicists, and administrators who conspired to reward, offered to reward, or actually rewarded the research participants. And from a sentencing perspective, being an accessory to a crime is in no way less blameworthy than being the main criminal perpetrator.

A reasonable question to ask is: Has the NPA ever pressed charges against anyone for transgressing section 60(4)? There are no reported cases to suggest that this ever transpired. However, this is no sure indication that it did not happen or that it cannot happen in future. It should also be noted that the NPA is independent of other branches of government, such as the Department of Health that administers the Act. This means that even if officials from the Department of Health—or the Minister of Health himself—openly favor giving rewards to research participants, the NPA still retains the discretion to prosecute instances of benefit sharing. The decision to prosecute should not be a political decision that is influenced by any government official, but purely a legal decision based on extant law. Accordingly, the question at the beginning of this paragraph warrants another question: Has anyone ever laid a criminal complaint relying on section 60(4)? In the absence of a complaint being filed, a criminal investigation and prosecution are unlikely. But should there ever be a disgruntled person who decides to file a criminal complaint, and an ambitious prosecutor is appointed to the case, mayhem may break loose. This may include extradition proceedings against researchers in foreign countries who have aided and abetted rewards for research participants in South Africa. (Whether extradition would be successful would depend on a number of factors, most prominently being whether there is a statutory provision similar to section 60(4) in the relevant foreign jurisdiction. If not, extradition would fail.)

Will the hypothetical fact that a South African government official spoke out in favor of benefit sharing in general, or even if they gave specific assurances to a person charged in terms of section 60(4) with giving rewards to research participants, make any difference? To sentencing, yes—but not to guilt. In other words, if these hypothetical facts are true, and the accused person is able to prove the facts in court, he or she will perhaps just get a light sentence, such as a warning—but still get a criminal record.

### III.E. De Minimis

But, what about the example of persons being given cookies when they donate blood to the South African National Blood Service? And in some cases, persons are even offered blankets, movie tickets, and an award ceremony! Are these not also rewards? And, if so, why are there no criminal prosecutions? The answer to the first question is affirmative: these are clearly rewards, although small in value. The answer to the second question is speculative. It may be because the NPA is not aware of the situation, or that they simply view this as being so trivial as not to justify their time, or a combination of both these factors. It should be noted upfront that non-enforcement of the law in one context (blood donation for transfusion) is problematic and is no reason in law for arguing that the same law ought not to be enforced in another context (donation of biospecimens for research). However, these examples do point to a possible argument against the enforcement of section 60(4) to benefit sharing situations that should be explored: The common law maxim *de minimis non curat lex—*the law does not concern itself with trifles.

Whether *de minimis* can come to the rescue of someone accused of rewarding research participants in contravention of section 60(4) will depend on the nature of the benefit sharing in question. On the one side of the spectrum of benefits, expressing gratitude to research participants in a published article would, we suggest, be insignificant from a criminal law perspective. However, on the other side of the benefit spectrum, offering to build a new clinic to a community, offering free medicine to research participants, or offering financial payments if the research culminates in a new medicine that is successfully monetized are certainly not insignificant. The following can be used as a yardstick: In a case of theft dating from 2002, where *de minimis* was pleaded, the court held that goods to the value of R59,66 were *not de minimis.*^35^ The present-day value would be around R160 (just under $10). In other words, a cookie is *de minimis*, a blanket and a movie ticket may be so, but any benefit over this amount would likely *not* be perceived by the South African courts as *de minimis*.

## IV. CONCLUSION: A POLITICAL SOLUTION

In sum, therefore, anyone advocating benefit sharing with research participants in South Africa needs to confront the legal reality that benefit sharing is unlawful and criminal in South Africa and if practiced it would expose all involved to prosecution. Needless to say, any benefit-sharing agreement that is held to be unlawful would also be legally void—in other words, an empty promise. The legion of ethical ramifications of ignoring the law should be obvious to the reader. The ethics discourse on benefit sharing with research participants in South Africa needs to grow out of its current naivety and face this legal reality.

If proponents of benefit sharing with research participants are serious about their aim, the only solution is a political one: The South African parliament must amend the National Health Act—in particular section 60(4). This will first entail public consultations and debates that will pit those in favor of retaining the altruistic paradigm against those who favor a shift away from altruism to a paradigm of benefit sharing. We suggest that such a public debate would be a good exercise. Neither of the competing paradigms are without their merits and demerits. Eventually, the democratically elected representatives of the people of South Africa must decide between these two paradigms. But, for now, section 60(4) is the law of the land—whether one likes it or not.

## Footnotes

1 NIH National Cancer Institute, *Glossary of Terms,* NCI Best Practices for Biospecimen Resources (Mar. 13, 2016), https://biospecimens.cancer.gov/bestpractices/got/.

2
Nikola Biller-Andorno & Alexander M. Capron, *Ethical Issues in Governing Biobanks: Global Perspectives* 2 (2016).

3 Anja Bedeker *et al., A Framework for the Promotion of Ethical Benefit Sharing in Health Research*, BMJ Global Health, Feb. 2022, 3.

4 See, for example: HUGO Ethics Committee, *HUGO Ethics Committee Statement on Benefit Sharing: Apr. 9, 2000*, 58 Clinical Genetics 364, 364 (2000).

5 Keymanthri Moodley & Anita Kleinsmidt, *Allegations of Misuse of African DNA in the UK: Will Data Protection Legislation in South Africa be Sufficient to Prevent a Recurrence?*, 21 Dev. World Bioeth. 125 (2021); S. Mahomed, et al., *The Role of Data Transfer Agreements in Ethically Managing Data Sharing for Research in South Africa,* 15 S. Afr. J. Bioethics & L. 26 (2022).

6 Alexander Morgan Capron, et al., *Ethical Norms and the International Governance of Genetic Databases and Biobanks: Findings from an International Study*, 19 Kennedy Inst. of Ethics J. 101 (2009); Bege Dauda & Kris Dierickx, *Benefit Sharing: An Exploration on the Contextual Discourse of a Changing Concept*, BMC Medical Ethics, Sep. 2013; Timothy Caulfield, et al., *A Review of the Key Issues Associated with the Commercialization of Biobanks*, 1 J. Law Biosci. 94 (2014).

7 D. Schroeder, *Benefit Sharing: It’s Time for a Definition*, 33 J. Med. Ethics 205, 208 (2007).

8 Kadri Simm, *Benefit-Sharing: A Look at the History of an Ethics Concern*, 8 Nature Reviews Genetics 496, 496 (2007).

9 Udo Schüklenk & Anita Kleinsmidt, *North–South Benefit Sharing Arrangements in Bio Prospecting and Genetic Research: A Critical Ethical and Legal Analysis*, 6 Dev. World Bioeth. 122, 124 (2006).

10 HUGO Ethics Committee, *supra* n. 4 at 364.

11 Bege Dauda & Kris Dierickx, *Benefit Sharing: An Exploration on the Contextual Discourse of a Changing Concept*, BMC Medical Ethics, Sep. 2013.

12 Lorraine Sheremeta & Bartha Maria Knoppers, *Beyond the Rhetoric: Population Genetics and Benefit-Sharing* in Accessing and sharing the benefits of the genomics revolution (Peter W. B. Phillips & Chika B. Onwuekwe eds., v. 11, 2007) at 171.

13 HUGO Ethics Committee, *supra* n. 4 at 366.

14
*Id.*

15 B. M. Knoppers, *HUGO Urges Genetic Benefit-Sharing,* 3 Public Health Genomics 88, 91 (2000).

16 Bedeker, et al., *supra* n. 3 at 3.

17
*Id.*

18
*Id.*

19 Academy of Science of South Africa & Department of Science and Technology, *Human Genetics and Genomics in South Africa: Ethical, Legal and Social Implications* (2018), https://research.assaf.org.za/bitstream/handle/20.500.11911/106/2018_assaf_ethical_genetics_genomics_consensus.pdf?sequence=1&isAllowed=y at 88–89.

20 Nchangwi Syntia Munung & Jantina de Vries, *Benefit Sharing For Human Genomics Research: Awareness and Expectations of Genomics Researchers in sub-Saharan Africa*, Ethics Hum. Res., Nov. 2020, at 17.

21 HUGO Ethics Committee, *supra* n. 4, at 365.

22 HUGO Ethics Committee, *supra* n. 4.

23 Bedeker, et al., *supra* n. 3, at 4.

24 Simm, *supra* n. 8.

25 Sheremeta & Knoppers, *supra* n. 11, at 171.

26 Bedeker, et al., *supra* n. 3, at 2; Michèle Ramsay, et al., *Ethical Issues in Genomic Research on the African Continent: Experiences and Challenges to Ethics Review Committees.* BMC Hum. Genomics, Aug. 2014.

27 Sheremeta & Knoppers, *supra* n. 11, at 171.

28 Donrich W. Thaldar & Bonginkosi Shozi, *Human Biological Material* in The Law of South Africa (W.A. Joubert ed., 3d ed. 2021) at para 15.

29
Lexico.com, https://www.lexico.com/definition/reward (last visited July 20, 2022).

30
Lexico.com, https://www.lexico.com/definition/thing (last visited July 20, 2022).

31 S. Afr. Const., 1996 § 10.

32 MEC for Education: KwaZulu-Natal v. Pillay 2008 1 SA 474 (CC) at 26 para. 53 (S. Afr.).

33 Khumalo and Others v. Holomisa 2002(5) SA 401 (CC) at 24 para. 27 (S. Afr.).

34 S v. Williams [1995] ZACC 6, 1995 (3) SA 632 (CC) at 30 para. 63 (S. Afr.): ‘our progress towards being a more humane and caring society’ (in the context of children’s rights); Port Elizabeth Municipality v. Various Occupiers [2004] ZACC 7, 2005 (1) SA 217 (CC) at 29 para. 37 (S. Afr.): ‘the constitutional vision of a caring society based on good neighbourliness and shared concern’ (in the context of illegal occupation of land), which passage has been quoted and relied upon in several subsequent Constitutional Court judgments; Khosa v. Minister of Social Development [2004] ZACC 11, 2004 (6) SA 505 (CC) at 40 para 65 (S. Afr.): ‘the constitutional commitment to developing a caring society’ (in the context of immigrants); Raduvha v. Minister of Safety and Security [2016] ZACC 24, 2016 (10) BCLR 1326 (CC) at 24 para 59 (S. Afr.): ‘our solemn undertaking as a nation to create a new and caring society’ (in the context of children’s rights).

35 S v. Tshabalala 2002 (1) SACR 605 (WLD) (S. Afr.).

